# Development of whole virion inactivated Kyasanur Forest Disease vaccine candidate and its preclinical safety and efficacy evaluation

**DOI:** 10.3389/fimmu.2026.1786057

**Published:** 2026-04-02

**Authors:** Raju Sunagar, Narayana Penta, Sreelekshmy Mohandas, Rekha Cheruvara, Rama Lakshmi Bonda, Praveen Algangula, Virendra Kumar Meena, Sathish Kumar, Sridevi Nimmagadda, Jitendra Narayan, Nivedita Gupta, Pragya Dhruv Yadav, Anand Kumar Kanakasapapathy

**Affiliations:** 1 Indian Immunologicals Ltd, Hyderabad, India; 2 Maximum Containment Facility, Indian Council of Medical Research-National Institute of Virology, Pune, India; 3 Electron Microscopy and Histopathology laboratory, ICMR-National Institute of Virology, Pune, India; 4 Division of Communicable Diseases, Indian Council of Medical Research, New Delhi, India; 5 Indian Council of Medical Research-National Institute of One Health, Nagpur, India

**Keywords:** KFD vaccine, KFDV challenge, KFDV lethal dose, KFDV preclinical toxicology, Kyasanur Forest Disease

## Abstract

Kyasanur Forest Disease Virus (KFDV) is a zoonotic tick-borne flavivirus that causes Kyasanur Forest Disease (KFD), a disease primarily found in India. KFD historically been confined to Karnataka state of southern India, however, in recent years, it has spread beyond its original endemic region, raising significant public health concerns. There are currently no KFD specific treatments or vaccines available. Rapid development of effective vaccine against KFD is urgently needed. We developed and evaluated a whole virion inactivated KFD virus vaccine candidate, adjuvanted with aluminum hydroxide [Al(OH)3] for its safety and efficacy in laboratory animal models. We used a well-characterized KFD virus strain and an established Vero cell platform to produce large-scale GMP-grade highly purified inactivated antigen. Two dose vaccination with the vaccine candidate (18 µg/dose) in Balb/c mice model elicited stronger IgG response and elevated neutralizing antibody as well as T cell cytokines responses and provided complete protection against KFDV challenge (100 LD_50_). The protected mice in vaccinated group showed reduced weight loss, controlled clinical symptoms and significantly low viral load in brain tissue. Passive transfer of KFD vaccine immune serum provided effective protection to naive mice against a lethal KFDV challenge (10 LD_50_). Single-dose and repeated-dose toxicity evaluation demonstrated that the vaccine is well tolerated, safe, and non-toxic. Our preclinical results support further advancement to human clinical trials and have obtained CDSCO approval for Phase I testing.

## Introduction

Kyasanur Forest disease virus (KFDV) is a tick-transmitted flavivirus that causes Kyasanur Forest disease (KFD) in humans (1, 2). The disease was first reported in 1957 in the Shimoga district of Karnataka, India. Since then, the disease has expanded beyond Karnataka, with reports from Goa, Kerala, Tamil Nadu and Maharashtra highlighting its geographic spread (3–5). Serological surveys have detected KFDV antibodies among the local population from Gujarat, West Bengal, and the Andaman and Nicobar Islands (2, 6–8).

KFD typically has an incubation period of 3–8 days, followed by a biphasic illness. The initial phase presents with fever, chills, frontal headache, and myalgia, followed by gastrointestinal and hemorrhagic manifestations, and in some cases, severe encephalitis and neurological complications (8, 9). The disease has a case fatality rate of 3–10% (4, 10), with 400–500 human cases reported annually (2). There is currently no approved antiviral treatment, and management is limited to symptomatic and supportive care.

To combat KFD, a formalin-inactivated tissue-culture vaccine was being used in endemic areas (11), with the regimen involving two primary doses a month apart, followed by a booster dose after 6–9 months, and subsequent boosters every five years (12). Vaccine effectiveness studies conducted in Karnataka indicated 62.4% effectiveness with two doses and 82.9% with an additional booster (13). Despite its widespread use, the vaccine faced criticism for limited efficacy, and by 2022, it was discontinued due to non-compliance with current regulatory standards and reduced public acceptance. Some studies suggest that genetic drift and strain variation in circulating KFDV compared to the vaccine strain may contribute to reduced efficacy (6, 7), indicating a critical need for a next-generation vaccine.

To address this gap, an inactivated whole virion vaccine was developed by the Indian Immunologicals Limited in collaboration with the Indian Council of Medical Research (ICMR)-National Institute of Virology, Pune. The inactivated whole-virion KFD vaccine candidate was formulated using Aluminum hydroxide as an adjuvant. Intramuscular administration in animal models demonstrated humoral and cell-mediated immune responses and provided protection against KFDV lethal challenge in mice. Comprehensive immunogenicity and safety evaluations were conducted in mice, rats, hamsters, and rabbits, and ready Phase I clinical trial to evaluate safety in humans.

## Results

### Vaccine strain selection

**Figure 1:**
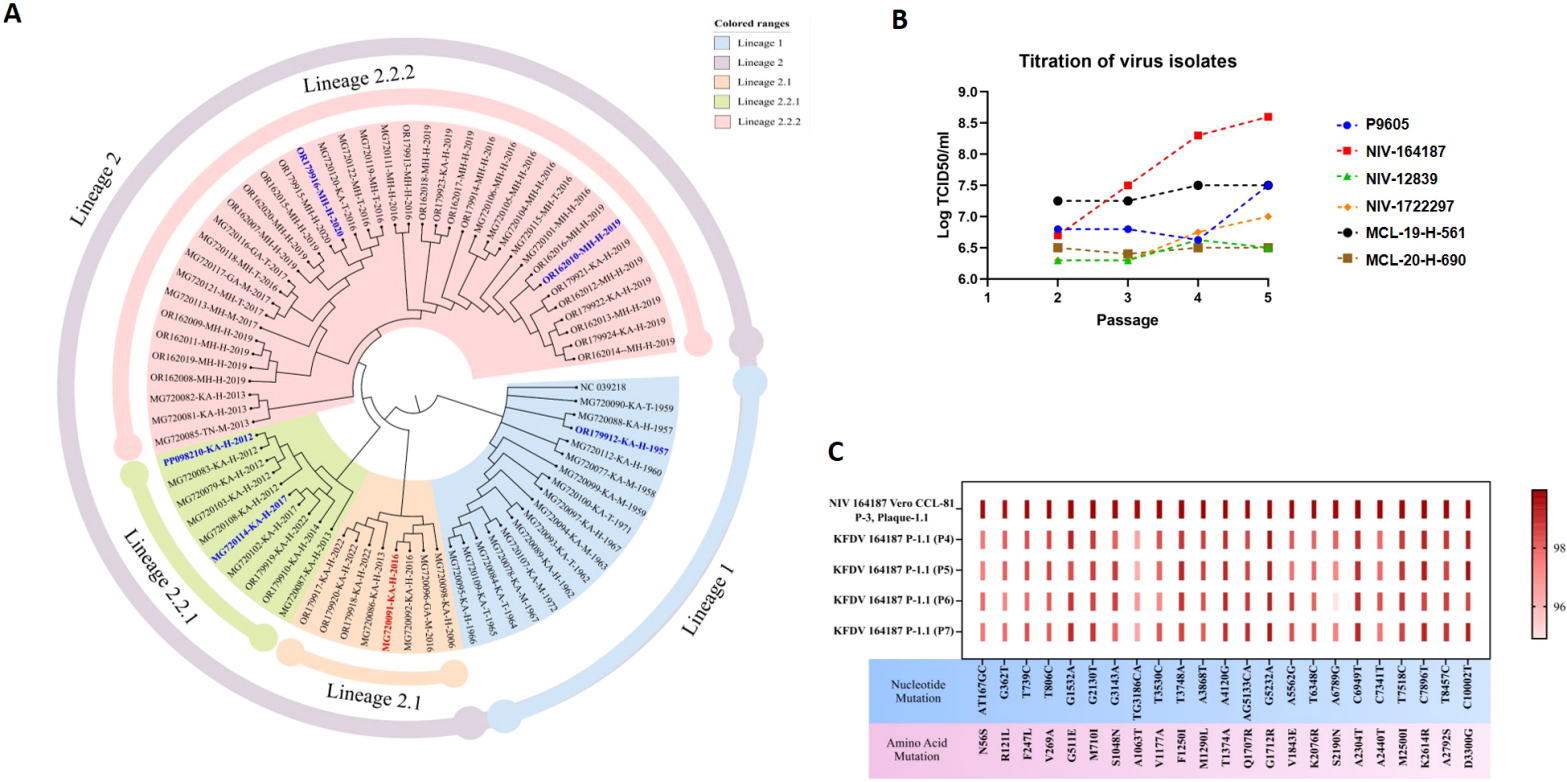
KFD vaccine strain selection. Phylogenetic analysis of whole genomes of 73 KFDV sequences using maximum likelihood method. The lineages are marked in color ranges and the isolates which are shortlisted for *in vitro* studies are highlighted in bold blue or red **(A)**. The titer of the shortlisted 6 KFDV isolates from passage 2 to passage 5 in Vero-CCL-81 cells **(B)**. Heatmap depicting the nonsynonymous mutations in the vaccine strain in comparison to the KFDV reference genome (NC_039218), showing absence of introduction of any additional mutations across passages in Vero-CCL-81 cells **(C)**. Each row represents a sample, and each column denotes a specific non-synonymous mutation, including its nucleotide substitution and corresponding amino-acid change. Red blocks indicate the presence of the mutation, while the dark-to-light color gradient reflects variation in mutation frequency or representation across samples.

Genomic analysis of KFDV strains (n=73) collected between 1957 and 2022 was conducted to assess strain diversity and antigenic variation. The whole genome analysis showed that the sequences of KFDV falls into 2 lineages, i.e., isolates of 1957-1972 in the lineage 1 and the isolates of 2006 to 2022 in lineage 2 (**Figure 1A**). KFDV strains (2006–2022) which falls into the lineage 2 differed from the lineage 1 (1957–1972) by an average of 2.76% and 0.8% in the nucleotide and amino acid divergence respectively. The envelope gene analysis also showed a variation of less than 0.8% in amino acid sequences of the lineage 2 sequences. Whole-genome analysis of KFDV sequences led to the shortlisting of seven isolates-six representing different sub-lineages of lineage 2 and one from lineage 1. These were low-passage isolates free from adventitious agents. (NIV-12839, NIV-164187, NIV-1722297, MCL-19-H-561, MCL-20-H-690 and P9605) and were adapted to Vero CCL-81 cells. Of the six, isolate NIV-164187 demonstrated the highest viral titer, reaching 10^8.6^ TCID_50_/ml (**Figure 1B**). Assessment of genetic stability across five passages revealed no detectable genomic variations (**Figure 1C**), confirming its stability during *in vitro* propagation. Given its high titer and genetic stability, NIV-164187 was selected for bulk production and further characterization. The selected KFDV vaccine strain was found to be neutralized by convalescent sera from KFD recovered cases. Accordingly, strain NIV-164187 was taken forward for GMP batch manufacturing and evaluation as a potential vaccine candidate.

### Production of KFD vaccine candidate

**Figure 2:**
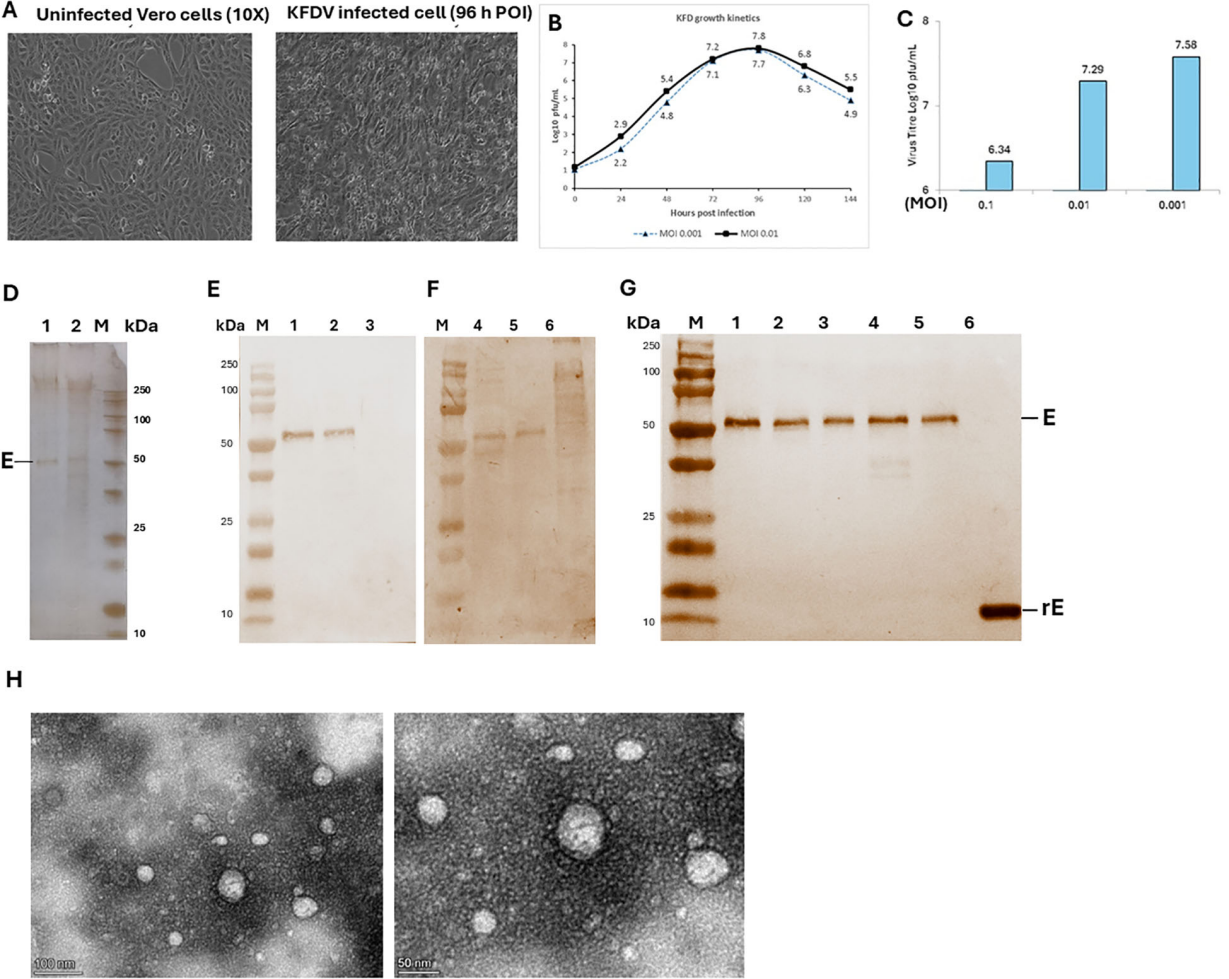
Characterization of KFD vaccine candidate. KFD virus growth kinetics and cytopathic effect (CPE) in Vero cells studied by infecting cells with 0.1, 0.01 & 0.001 MOI of KFDV virus **(A)**. Samples were collected at 24 hours intervals and were subjected to KFDV titration **(B)**. Comparison of KFDV yield against various MOI post-24h infection **(C)**. Protein composition of KFD vaccine drug substance by SDS-PAGE and silver staining, Lane 1: Purified KFDV; lane 2: Inactivated KFDV antigen; lane M: Protein marker (Biorad-1610374) **(D)**. Western blot analysis of purified inactivated KFD vaccine drug substance (lanes 1&4: batch-3; lanes 2&5: batch-4) detected using KFDV specific monoclonal antibody clone 5D3.A1.F3 **(E)** or convalescent human sera **(F)**, lane M-Protein marker (Biorad-1610374). KFDV drug substance from five production batches (lane 1-5), along with *E. coli* expressed recombinant envelope protein ectodomain [Nordic Biosite, BTF38GD8 (lane 6)] detected using KFDV specific monoclonal antibody clone 5D3.A1.F3 **(G)**. Representative electron micrograph of purified KFDV candidate vaccine at a scale bar:100nm (right) and 50 nm(left) **(H)**.

KFDV seed virus NIV-164187 was propagated in a highly characterized GMP Vero cell platform, to produce the master and working virus bank. To manufacture the purified bulk antigen, the virus propagation and purification process to produce purified bulk was performed facility using cell stacks in the bio-safety level 3. The master and working virus bank was well characterized for identity, sterility, titer, presence of Mycoplasma/adventitious agents. In the absence of specific guidelines for the KFD vaccine, the candidate vaccine was characterized in accordance with the WHO guidelines for the JE vaccine (WHO-TRS_963_Annex_1.pdf, 2007). Growth kinetics analysis of the KFDV (adapted to GMP Vero cell platform) in Vero cells showed a peak titer of 7 log_10_ PFU/mL by 90-96 hours of post-infection (**Figure 2A**) at multiplicities of infection of 0.01 to 0.001 (**Figures 2B, C**). To produce pilot-scale KFD vaccine for preclinical evaluation, the virus was propagated in the cell factory system (3xCS10) and the virus was inactivated with the β-propiolactone (BPL). Inactivation kinetics were assessed under varying conditions and concentrations, with samples collected at multiple time points to monitor cytopathic effects. The inactivation procedure was carried out for five consecutive batches to verify complete viral inactivation while maintaining antigen stability. Inactivated whole-virion antigen was purified using depth filtration followed by optimized chromatography (**Figure 2D**), the purified antigen was further characterized for identity using KFD virus specific monoclonal antibody clone 5D3.A1.F3 (in-house reagent) as well as convalescent sera (**Figures 2E, F**). The purified inactivated antigen bulk of the vaccine candidate showed the reactivity with envelope protein (~54 kDa) (**Figure 2G**) and the monoclonal antibody detected the recombinant envelope (ectodomain) (**Figures 2G**, lane 6). Transmission electron microscopy (TEM) analysis of KFD vaccine revealed that the inactivated and purified virus particles remained intact, appearing as spherical virions with diameters typically ranging from 40 to 60 nm (**Figure 2H**).

The inactivated, purified whole-virion antigen, produced from five consistent batches with an average protein yield of approximately ~10 mg/L of culture. Following the removal of process-related impurities—including host cell proteins (HCP), host cell DNA (HCD), bovine serum albumin (BSA), residual BPL, residual endonuclease, and media components. The sterile inactivated KFDV drug substance (DS) was prepared and stored at 2–8 °C until further use.

### Vaccine formulations and immunogenicity studies

The adjuvants aluminum hydroxide (Al(OH)_3_) gel and aluminum phosphate (AlPO4) were used to blend the inactivated whole virion KFDV antigen at different antigen concentrations (i.e. 6, 12, 18, and 24 µg per dose). The final blend of KFD vaccine candidate consisted of inactivated KFDV antigen, Al(OH)3 or AlPO4 as an adjuvant (1mg/mL), thiomersal as a preservative (0.01%/mL), and phosphate buffered saline (PBS) pH 7.3 ± 0.2 as a diluent.

**Figure 3:**
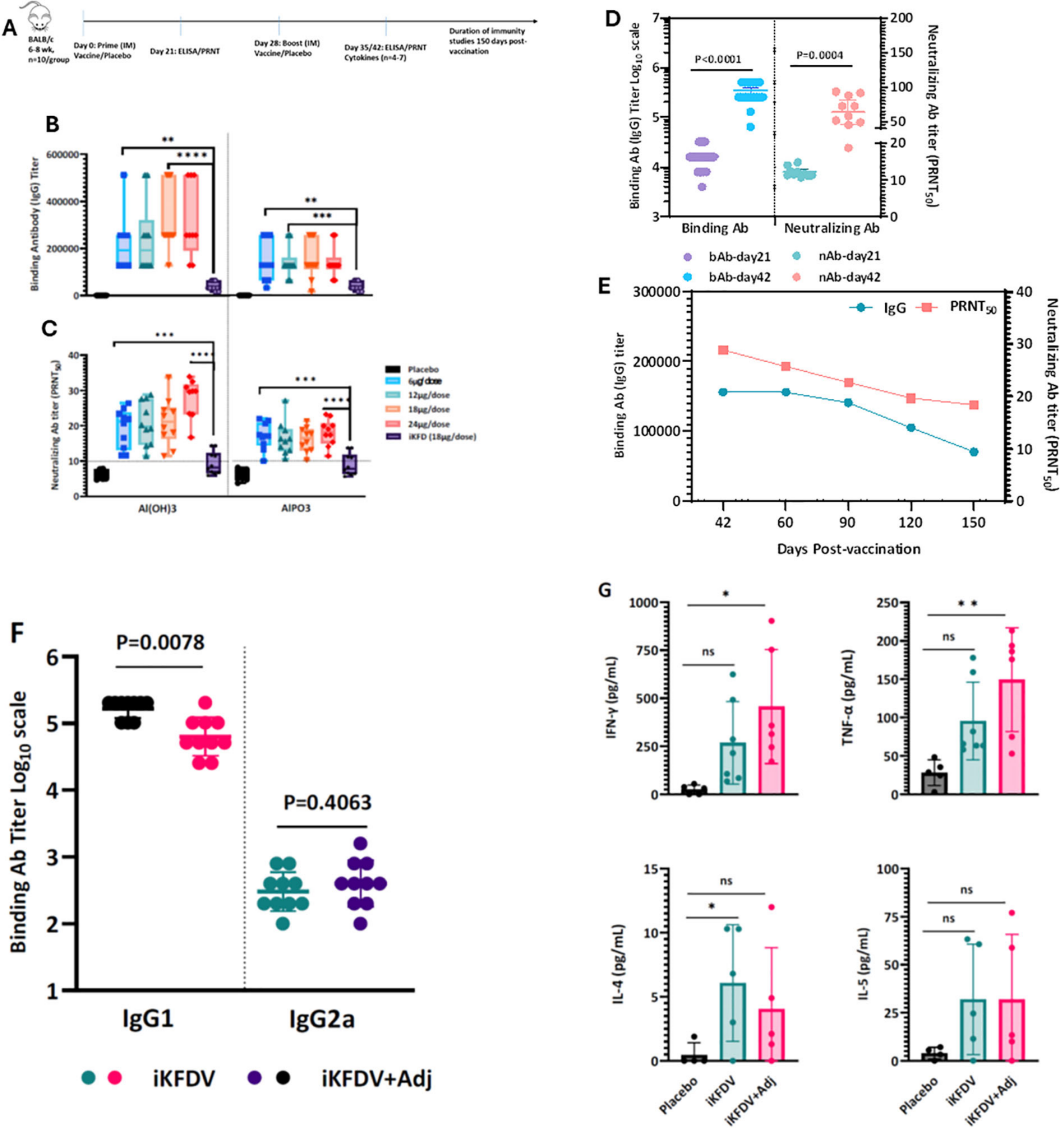
Candidate vaccine elicits robust humoral and cell mediated immune response in mice. Schematic diagram of immunization regime mouse experiment **(A)**. Six to eight-week-old BALB/c male and female mice were immunized on day 0 and boosted on 28 days with various doses of KFD vaccine or placebo (n = 10) via intramuscular route. Binding and neutralizing antibody responses at 2 weeks post-vaccination (day 42) in sera of immunized mice at various doses of KFD vaccine or placebo. Binding antibody levels against Inactivated whole virion KFDV **(A)** were measured by ELISA. Neutralizing activity of immune serum against wild type KFDV was measured by PRNT_50_**(B)**. Binding and neutralizing antibodies were measured in serum samples collected from mice immunized with either a prime-only or prime-boost regimen of the KFD vaccine or place **(C)**. Longitudinal KFDV specific-binding and neutralizing antibody responses **(D)** measured by ELISA and PRNT_50_ respectively. The profile of IgG1 and IgG2a titer measured by ELISA, using sera collected from day 42 (14 days post-booster), from BALB/c mice **(E)**. Endpoint titer of respective immunoglobulin sub classes obtained from Placebo were taken as baseline. Error bars represent mean G SEM. Secreted cytokine production by splenic T cells was measured by ELISA **(F)**. Splenocytes were harvested from a subset of mice (n = 4-6) at 2 weeks post-vaccination and re-stimulated with inactivated KFDV whole virion for 72 hrs **(G)**. Statistical significance for binding and neutralizing antibody titers was performed with parametrict unpaired t test for IgG, Wilcoxon matched-pairs signed rank test for IgG1 and IgG2. For cytokine response performed with non-parametrict Mann-Whitney t test.: **P=0.01; ***P=0.0005; ****P=<0.0001.

The immunogenicity of the KFD vaccine in BALB/c mice was assessed following intramuscular administration on Days 0 and 28. The study design included multiple antigen doses formulated with either Al(OH)_3_ or AlPO_4_, a KFDV antigen-alone group (18 µg/dose), and a placebo group containing adjuvant and excipients without KFDV antigen **Figure 3A**. The binding and neutralizing antibody response assessed on day 42 post-vaccination demonstrated that the adjuvanted vaccine formulations elicited stronger immune response compared to inactivated KFDV whole virion alone. Both the Al(OH)_3_ and AlPO4 adjuvanted formulations induced dose-dependent binding and neutralizing antibody responses. Furthermore, the Al(OH)_3_ formulated antigen elicited a stronger immune response compared to the AlPO_4_ formulation (**Figure 3B**). The 18 µg dose group showed higher binding antibody titers compared with the lower-dose groups (6 & 12 µg/dose) GMT 222861 vs 157937 &174012 respectively. The titers were also broadly comparable to those observed in the higher antigen group (24 µg/dose) GMT 222861 vs 276495 (**Figure 3C**). Based on this profile, the18 µg dose formulated with Al(OH)3 was selected for further assessment in subsequent studies. A single vaccine dose elicited moderate antibody levels, while a significant increase in both binding and neutralizing antibody titers was observed after the booster dose (**Figure 3D**).

The immune sera were further assessed for envelope ectodomain–specific antibodies, which is primary immunogen responsible for eliciting protective immunity. As expected, KFD vaccine immune sera demonstrated recombinant ectodomain specific IgG response, although the magnitude was lower than that observed with the whole-virion antigen (**Supplementary Figure 1**). The duration of antibody response was assessed up to 150 days post-vaccination, all vaccinated mice maintained the seroconversion and exhibited both binding and neutralizing antibodies (nAb)s with a geometric mean titer (GMT) of 70661 and PRNT_50_ 18 respectively (**Figure 3E**). Both binding and neutralizing antibody levels were sustained for up to 90 days post-vaccination, with only a marginal decline observed over time. Immunoglobulin subclasses (IgG1, IgG2a, and IgG3) were analyzed on day 42 to evaluate the Th1/Th2 polarization and it was found that higher IgG1 response in both Al(OH)3 formulated antigen and antigen alone groups compared to IgG2a indicative of Th2 bias (**Figure 3F**). Having observed a robust antibody response in vaccinated mice, the cell mediated immune (CMI) response was examined. *Ex vivo* stimulation of splenocytes with inactivated whole KFDV antigen resulted in a significant induction of Th1 associated IFN-γ or TNF-α cytokines in animals immunized with the antigen formulated in Al(OH)3 compared with placebo group (**Figure 3G**). In contrast, splenocytes from animals vaccinated with antigen alone produced modest levels of Th1 associated IFN-γ or TNF-α as well as Th2 associated IL-4 and IL-5,relative to splenocytes from placebo-treated mice.

### Candidate vaccine protects Balb/c mice against lethal KFDV challenge

**Figure 4:**
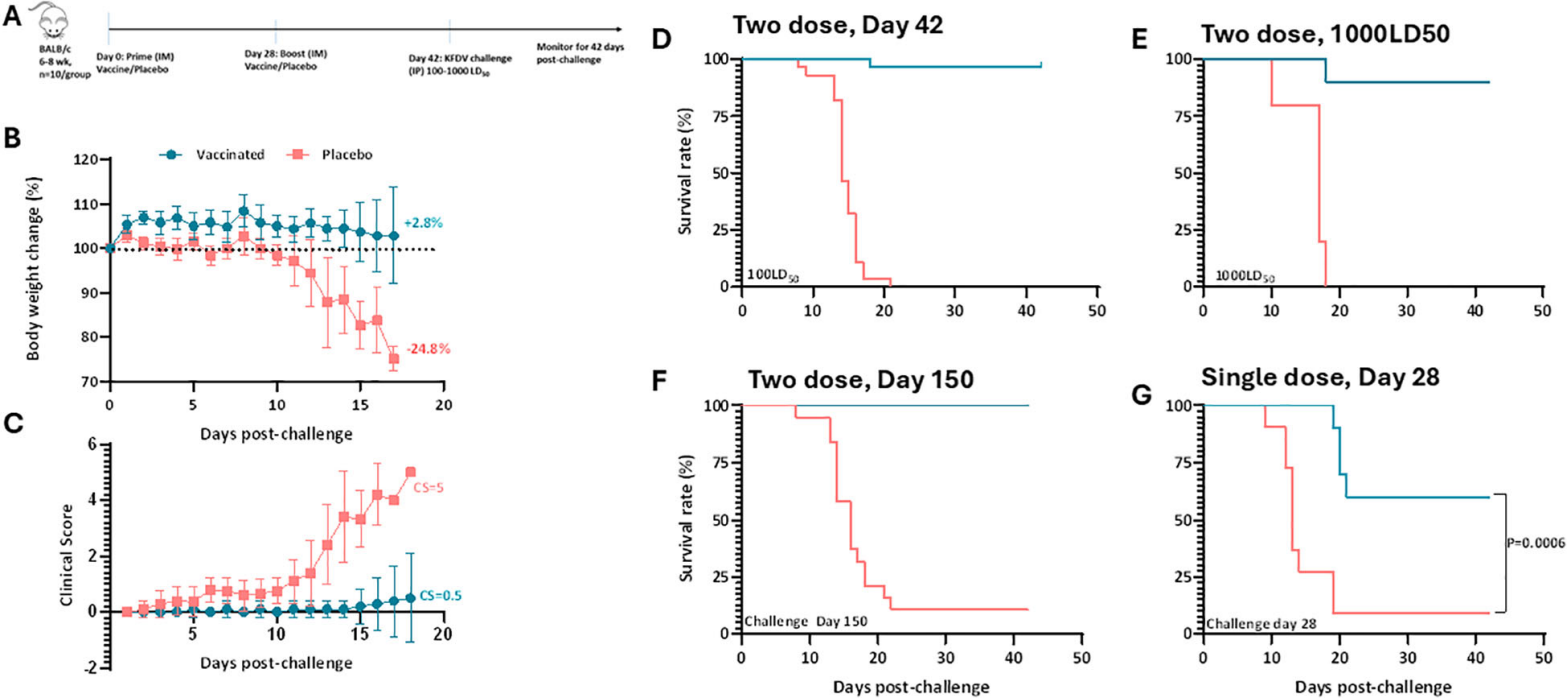
Protective Efficacy of KFD vaccine against wild type KFDV lethal challenge. Schematic diagram of immunization regime mouse experiment **(A)**, six-eight-week-old BALB/c male and female mice (n=10) were immunized with two doses of KFD vaccine or placebo as explained in **Figure 3**, mice were challenged on day 42 post-vaccination. Challenged animals were monitored for Weight loss **(B)** and clinical signs **(C)** following lethal challenge with wild type KFDV with 100 LD_50_ **(D)** or 1000 LD_50_ **(E)** or Subset of mice challenged on day 150 post-vaccination with 100 LD_50_ **(F)**. Group of mice immunized with single dose vaccination subjected for wild type KFDV challenge on day 28 **(G)** post-vaccination, mice were monitored for survival for 42 days. Statistical significance for survival curves was analyzed using the Logrank (Mantel-Cox) test and for body weights using unpaired T tests.

The protective efficacy of the KFD vaccine was assessed by challenging vaccinated mice with 100–1000 LD_50_ of the KFD virus. To determine the LD_50_ of the challenge virus, groups of BALB/c mice (n=10) were inoculated intraperitoneally with varying doses of KFDV ranging from 0.3 to 6.3 log_10_ PFU. Animals displayed dose dependent clinical manifestations and mortality typically occurred around 9–11 dpi. Mortality rates varied with dose: 20% at 0.3 log_10_ PFU/mouse, 50% at 2.3 log_10_ PFU/mouse, and 100% at 4.3 log_10_ PFU/mouse. The lethal dose (LD_50_) of the KFD virus determined in BALB/c mice was found to be 2.38 log_10_ PFU (**Supplementary Figure 2**). In protection experiments using a 100 LD_50_ challenge, mice immunized with the 18 µg dose achieved complete protection against the high dose lethal challenge, whereas those receiving lower doses (6 µg and 12 µg) exhibited partial protection (80%) (**Supplementary Figure 3**). Notably, mice vaccinated with the 18 µg dose presented neither weight loss nor signs of clinical disease, confirming complete protective efficacy (**Figures 4A, B**). In contrast, placebo-treated animals developed severe clinical symptoms (**Supplementary Table 1**), experienced up to 25% body-weight loss, and succumbed to infection by day 20 post-challenge (**Figure 4B**). When the challenge dose was increased to 1000 LD_50_, all placebo-treated mice exhibited a 20–30% reduction in body weight and succumbed to Kyasanur Forest disease virus infection by day 19, whereas 90% of vaccinated mice survived (**Figure 4C**). To assess the durability of protection, the challenge (100 LD_50_) was delayed until 20 weeks post-vaccination. Placebo animals succumbed to infection with mean time to death (MTD) of 18 days, while complete (100%) protection was observed in vaccinated mice (**Figure 4D**). These findings prompted us to evaluate the vaccine potency in a simulated pandemic scenario by assessing the efficacy of a single dose regimen. Accordingly, following single dose vaccination mice were challenged at two- and four-weeks post-vaccination. At four weeks post-vaccination, 50% of vaccinated mice were protected, whereas 90% of placebo animals succumbed to infection, exhibiting approximately 25% body weight loss and an MTD of 18 days (**Figure 4E**, **Supplementary Figure 4**). In contrast, challenge at two weeks following a single dose did not provide significant protection; vaccinated mice showed marked weight loss comparable to unvaccinated controls, with only a marginal (two-day) delay in MTD (**Supplementary Figures 5A, B**).Indicating that a minimum of four weeks post-vaccination is required for the development of protective immunity.

### KFD vaccination controls the KFDV infection in the brain

**Figure 5:**
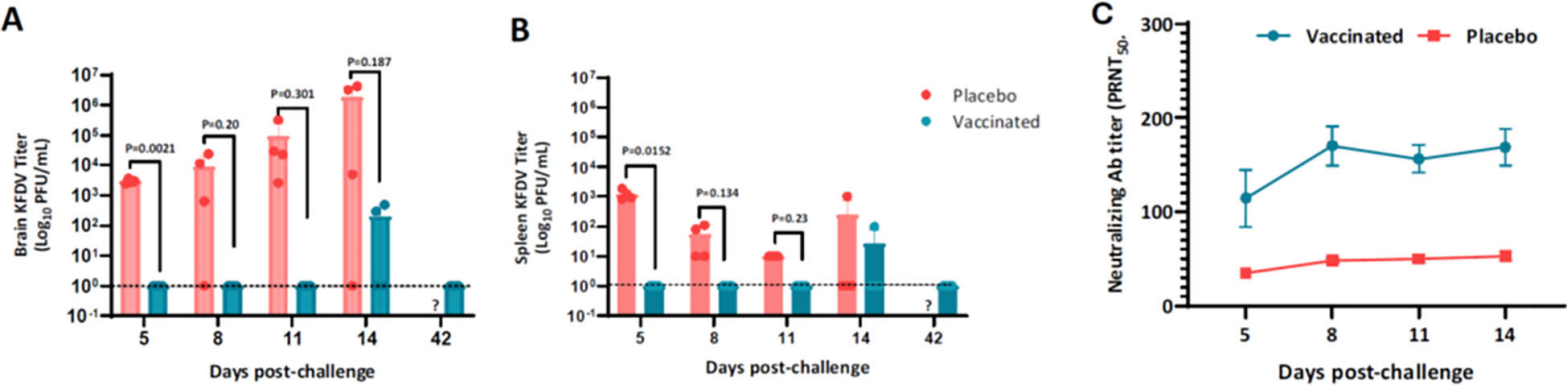
Protection mediated through viral clearance. Six-eight-week-old BALB/c male and female mice were immunized as explained in **Figure 3** challenged with KFDV 100 LD_50_. Viral load (titer/mL) from brain **(A)** and spleen **(B)** were determined on days 5, 8, 11, 14, and 42 post-challenge (n=4). The values represent the average viral titer of four mice sacrificed at each time point ± SD and are from a single experiment. Similar results were obtained in two independent experiments. Mice were monitored daily throughout the study the scores following KFDV challenge are presented **(C)**. On days 5, 8, 11, and 14 KFDV neutralizing antibody (PRNT_50_) in sera evaluated. Statistical analysis was performed with unpaired t-test with Welch’s correction.

To further assess the KFD vaccine-induced protective immunity, blood, brain, and spleen samples from subset of animals (n=4) were examined for viral load following challenge with KFDV. Mice in the placebo group showed increase in the KFDV viral load in brain ~3.5 log_10_ PFU to ~6.5 log_10_ PFU from days 5 to 14 post-challenge (**Figures 5A, B**), with the peak viral burden on day 14 in this model. Notably, it was not until day 14 post-challenge that placebo mice began to die (**Figures 4B–G**). Strikingly, none of the vaccinated mice showed no detectable infectious virus either in brain or spleen till day 11 post-challenge. On day 14, two of four mice exhibited low viral titers (2 log_10_) in the brain, with one also showing viral presence in the spleen. Notably, by day 42 the virus was cleared from both the brain and spleen in vaccinated mice. We hypothesized that anamnestic response would be significantly elevated in vaccinated mice following KFDV challenge. Indeed, post-challenge assessment of nAb in vaccinated mice displayed 2–4 times higher titer than those in placebo-treated controls (**Figure 5C**), suggesting that nAbs elicited post-challenge may contribute substantially to the enhanced protection observed in vaccinated mice.

### Passive transfer of KFD vaccine hyperimmune sera protected mice against lethal KFDV challenge

**Figure 6:**
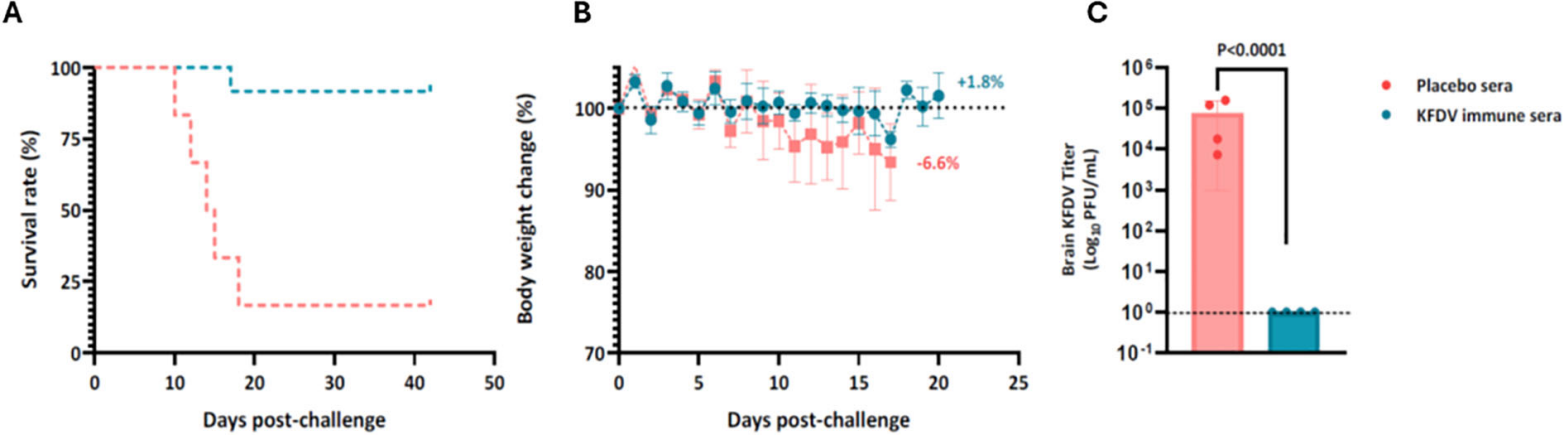
Passive transfer of immune sera induced by a KFD vaccine protects mice against lethal KFD virus challenge. Six-eight-week-old mice were treated IP with 500 µl of pooled sera from mice that were immunized with two doses of either KFD vaccine or placebo and challenged 24 hours later with KFDV 10 LD_50._ Mice were monitored for survival **(A)**, and weight-loss **(B)** for 42 days. On 11 dpi, subset of mice (n=4 per group) was euthanized for KFD virus load in brain tissues **(C)**. Error bars represent the standard deviation. Statistical significance for survival curves, body weights and brain viral titer using the Mantel–Cox test, the unpaired t-test and t-test with Welch’s correction respectively.

The protective capacity of passively transferred KFD vaccine immune sera and placebo immune sera was evaluated to naive mice. Pooled immune sera from mice vaccinated with two doses of KFD vaccine or placebo groups (n=20 per group). The KFDV specific binding antibody (IgG) and the neutralization antibody level of the pooled sera was determined to be GMT ~256000 and ~23, respectively. BALB/c mice (n = 10 per group) were administered by intraperitoneal (IP) route with 500 µl of pooled sera 24 h prior to challenge with 10 LD_50_ of KFDV and mice were monitored for 42 days. Following challenge, subset of mice (n = 6) was monitored for survival and clinical signs for up to 42 days. Sera derived from KFD vaccine showed a protective efficacy of 83.3% (5 out of 6 mice survived) with no weight loss or signs of disease were observed in surviving mice following KFDV challenge (**Figures 6A, B**). In contrast, only 16.6% of the control animals survived the KFDV challenge (1 out of 6), all nonprotected mice displayed weight loss, ruffled fur, hunched posture, lethargy, ataxia, or neurological symptoms (**Supplementary Table 1**). High KFDV loads (10^4^ to 10^7^ TCID50/g) were detected in the brain tissues from mice administered with placebo immune sera on 12 days post-challenge. In contrast, KFD virus was not detected in brain tissue of any of the mice administered with KFD vaccine immune sera (**Figure 6C**). Indicating, the immune sera were able to protect mice against lethal KFDV challenge by controlling weight-loss, virus dissemination and morbidity, and mortality.

### The KFD vaccine induces cross-neutralizing antibodies but does not exhibit cross-reactivity with other flaviviral pathogens

**Figure 7:**
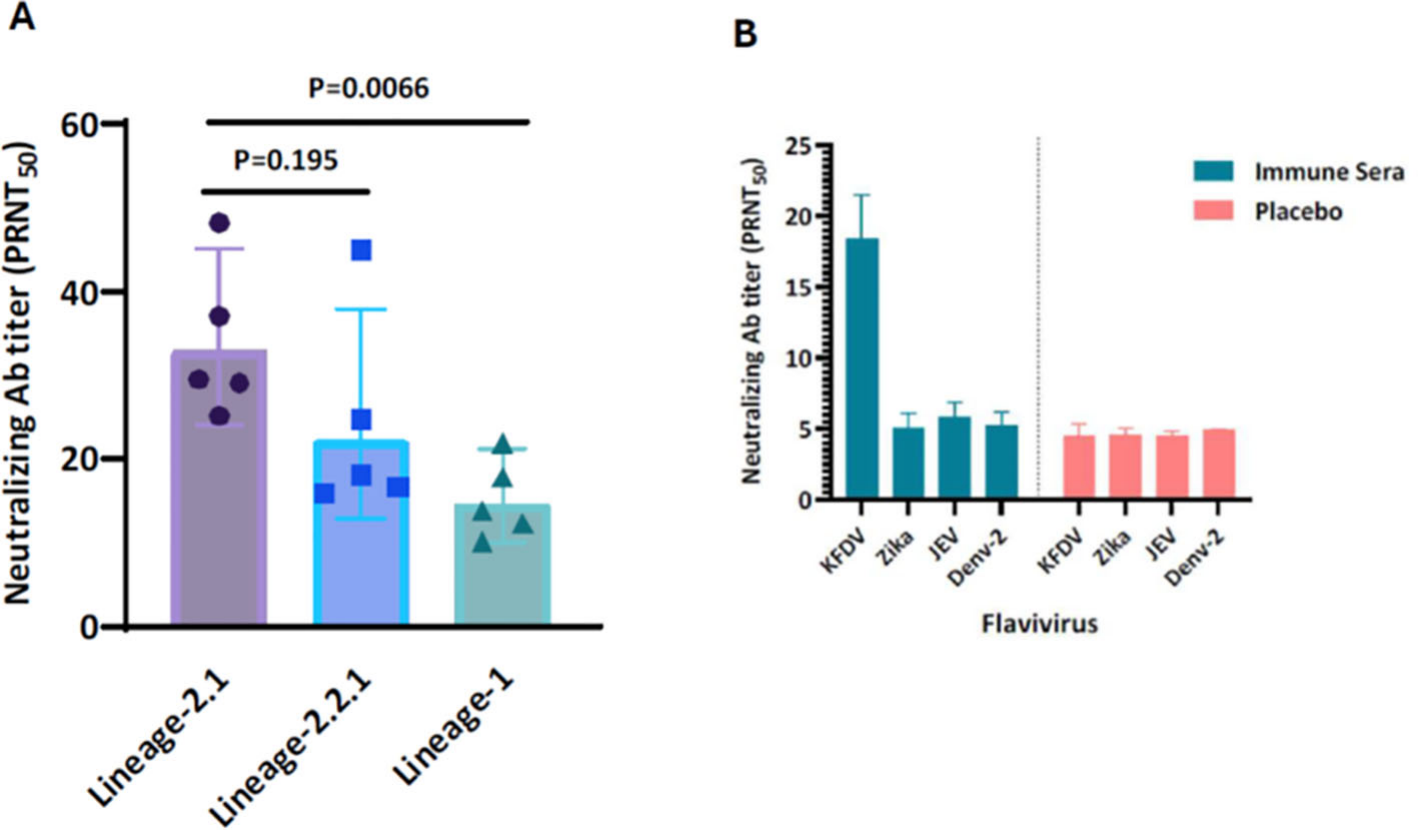
Cross neutralization and cross reactivity of KFD vaccine immune sera. The pooled sera from mice (n=10-12) that were immunized with two doses of either KFD vaccine or placebo was evaluated for the cross-neutralizing activity against heterologous KFD virus circulating isolates by microneutralization test **(A)**. The KFD vaccine immune response was further assessed for the cross-reactivity against non-KFD viruses like Zika, JE and Dengue-2 virus by PRNT_50_ **(B)**. The sera were diluted with DMEM medium for both vaccine and placebo samples, neutralized with individual virus having 10^3^ PFU/ml. The virus-sera mixture is added onto the respective cells and incubated for 4 days for plaque formation.

To evaluate the breadth and durability of the neutralizing antibody responses, immune sera from vaccinated mice were tested against circulating KFDV isolates. KFD vaccine immune sera demonstrated comparable neutralizing antibody responses against lineage 2.1 (NIV-164187) and lineage 2.2.1 (NIV-12839), while showing a 0.5-fold (P = 0.0066) reduction in neutralization titers against the lineage 1 strain (P9605) of KFDV. These findings suggest that the vaccine elicits broadly reactive antibodies (**Figure 7A**). Given that KFDV is a flavivirus, there is increasing scientific interest in evaluating whether neutralizing antibodies generated during KFDV infection exhibit cross-reactivity with other flaviviruses such as ZIKV, JEV, and DENV. These viruses share structural features—particularly in the envelope (E) protein, which is a major antigenic determinant and the primary target of neutralizing antibodies. Cross reactivity assessment of KFD vaccine immune sera demonstrated that neutralizing antibodies generated in response to KFD vaccine are highly specific to KFDV and do not exhibit cross-neutralizing activity against other flaviviruses such as Zika virus (ZIKV), Japanese Encephalitis Virus (JEV), or Dengue virus serotype 2 (DENV-2) (**Figure 7B**). The PRNT_50_ values for the non-KFD viruses-Zika, JEV and Dengue-2 were low (5.1, 5.9 & 5.2 respectively) and largely indistinguishable from those of the placebo control sera (4.5, 4.5 and 4.9). Strongly indicating that minimal reduction in plaque counts reflect non-specific background activity rather than true immune-mediated neutralization. The mean PRNT_50_ values against in the KFDV was found to be approximately ~4-fold higher than that of the placebo group. This substantial difference highlights the presence of strong virus-specific neutralizing antibodies in the immunized individuals.

### The protective efficacy of KFD vaccine correlated with neutralizing antibody titers

**Figure 8:**
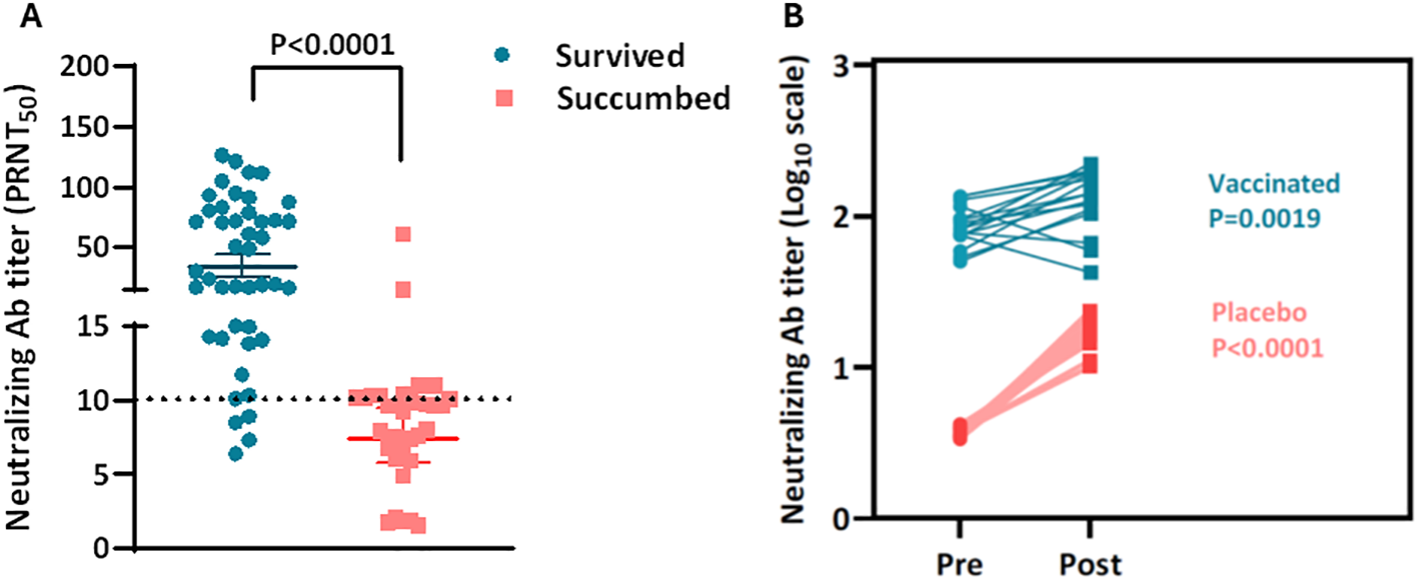
The protective efficacy of KFD vaccine was correlated with neutralizing antibody titers. The neutralizing antibody titers (PRNT_50_) on day 42 following two-dose vaccination, comparing protected and unprotected mice as correlates of protection **(A)**. Comparison of the neutralizing antibody titers **(B)** in paired sera of mice collected before challenge and 8 days after KFD virus challenge. Samples from the same mouse are connected with a line. Mice immunized with two doses of either KFD vaccine or placebo were pooled for analysis. A unpaired t test with Welch’s correction was used for statistical analysis.

Several studies have examined nAbs as correlates of protection (CoP) in flaviviral vaccination. Immune CoP were evaluated based on the capacity of circulating nAb to protect mice against severe disease followed by death of animal against KFDV challenge. The distribution of the individual mice based on their level of circulating nAb titer at one week pre-challenge demonstrated a significant inverse relationship between level of circulating nAb and mortality (p = 0.0001) (**Figure 8A**). Except four of forty-three mice that had a PRNT_50_ antibody level of approximately ~10 or higher survived. While the majority of mice with a PRNT_50_ titer around or below 1:10 succumbed to KFDV infection, two mice from the vaccinated group, despite having higher neutralizing antibody levels, also succumbed to the infection. We further compared the serum neutralization antibody titers of mice before and after the KFDV challenge. Control mice showed an average of 4.5-fold (P<0.0001) increase in neutralizing antibody day 8 post-challenge, whereas the vaccinated animals presented only 1.5-fold (P = 0.0019) elevation in corresponding neutralizing antibody titers (**Figure 8B**). Indicated that there was no evidence of an anamnestic response following KFDV vaccination in mouse.

### Safety evaluation of KFD vaccine formulation

An extensive safety evaluation for the KFD vaccine candidate formulation was performed as per New Drugs & Clinical Rules, 2019 and WHO guidelines (WHO guidelines on non-clinical evaluation of vaccines. WHO Technical Report Series, No.927, 2005). Acute and repeated-dose toxicity studies were conducted in appropriate animal models to assess the safety profile of the vaccine candidate. In the acute toxicity study, the inactivated KFD vaccine at twice the absolute human dose (2x HD) was administered as a single dose via intramuscular (intended route) and subcutaneous (alternative route) injection in Swiss Webster mice and Sprague Dawley rats. Animals were monitored throughout the observation period for clinical signs, mortality, body weight changes, and rectal temperature. No morbidity or mortality, abnormal clinical signs was observed in any of the placebo or treatment groups. Furthermore, no treatment-related statistically significant differences were observed in body weight change, or rectal temperature between the treatment, placebo, and negative control groups in either sex. Gross pathological examination did not reveal any treatment-related systemic abnormalities in the animals.

In the repeated-dose toxicity study, the vaccine was administered intramuscularly at three time points (n+1 = 3; where n is the number of doses in clinical regimen) i.e., on Days 0, 14, and 28 at three dose levels (0.5x, 1x, and 2x of the Human Dose) in Sprague Dawley rats and New Zealand White rabbits. Animals were monitored for clinical signs, body weight changes, feed consumption, and rectal temperature. No treatment-related clinical signs were observed, and no significant changes were detected in body weight, feed intake, or rectal temperature. Ophthalmological examinations, hematological parameters, and serum biochemical analyses did not reveal any significant alterations. Peripheral blood smear examination showed no morphological abnormalities, and bone marrow smear analysis revealed no cellular or morphological changes across all groups. Histopathological examination at the injection site demonstrated mild inflammatory changes characterized by the presence of macrophages, lymphocytes, polymorphonuclear cells, or a combination of these cells, along with congestion, neovascularization, and/or fibroplasia. These findings were considered consistent with localized inflammatory responses attributable to the adjuvant rather than the vaccine antigen. Overall, the vaccine candidate was well tolerated in both acute and repeated-dose toxicity studies and demonstrated a favorable safety profile, supporting its suitability for further clinical development.

### KFD vaccine is immunogenic in rabbits, rats, and hamsters

The immunogenicity and tolerability of clinical batch samples of KFD vaccine were evaluated in Wistar rats and New Zealand White rabbits with a full human dose (N + 1) regimen. Repeated doses of candidate vaccine 18ug/dose per animal was administered IM on days 0, 14 and 28. Serum samples were collected 21 days post-primary or pre-prime booster immunization and KFV specific IgG and neutralizing antibody response were evaluated. Repeated dose administration elicited significantly higher IgG responses GMT 13561; 4525 in rats and rabbits respectively (**Supplementary Figure 6A**). Consistent with the binding antibody response, KFDV vaccination elicited substantial increases in the KFDV specific neutralizing antibodies PRNT 20 and 18 in, rats and rabbits (**Supplementary Figure 6B**). We evaluated the immunogenicity of candidate vaccine in Syrian Hamsters following vaccination significant KFDV-specific IgG (GMT 8600) and neutralizing antibody (PRNT_50_ 23) responses were detected in most of the vaccinated hamsters (**Supplementary Figure 6C**).

Discussion. Although KFD has posed a significant public health challenge for over six decades and its geographic spread continues to increase, effective countermeasures remain lacking. The recent expansion of the KFDV-endemic region (4, 5), coupled with a rising number of KFD cases (2), highlights a growing public health threat (14). This underscores the urgent need for improved interventions, including the development of more effective second-generation vaccine against KFDV. Here we developed an inactivated whole-virion KFD vaccine formulated in Aluminum hydroxide adjuvant and characterized its protective efficacy in a KFDV mouse model.

Indian Immunologicals Limited in collaboration with ICMR-NIV developed a whole-virion inactivated KFD vaccine candidate. The vaccine strain NIV-164187 was isolated from a human serum sample collected during the 2016 outbreak in Belgaum, Karnataka. This strain represents KFDV lineage 2, which includes isolates reported between 2006 and 2022 (15). The virus was propagated in GMP-grade Vero cells in BSL-3, meeting WHO guidelines. The formalin-inactivated KFD vaccine, which was discontinued due to residual formalin-related side effects, was a major factor contributing to vaccine hesitancy (16). Moreover, formalin inactivation can compromise antigenicity and reduce vaccine efficacy (11, 17, 18). In contrast, we utilized β-propiolactone (BPL), a potent and safe inactivator that maintains the structural integrity of the whole virus while eliminating infectivity and stimulates strong B and T cell immune responses, and holds promise for improving vaccine acceptance and coverage (19–21).

It is established that the efficacy of a vaccine is improved by addition of adjuvant to the antigen. Accordingly, the KFD vaccine with (Al(OH)_3_) formulation produced higher antibody titers compared to the (AlPO4) formulation. The vaccine candidate also showed dose-dependent immune response, eliciting both binding and neutralizing antibodies (**Figures 3B, C**). The detection of anti-envelope antibody against recombinant envelope antigen indicates that, the current vaccine formulation induced the immune response, which is shown to be crucial in controlling flavivirus infections (22–24). The clinical and virologic profiling of KFDV patient samples findings demonstrated a strong activation of both T and B cells, with a particularly pronounced CD8+ T cell response (20, 25). Our vaccine candidate demonstrated a balanced induction of Th1 and Th2 cytokines (**Figures 3F, G**). Although the immune response is Th2 skewed, characteristic of alum-adjuvanted formulations, it still elicits sufficient cell-mediated immunity, which is critical for effective viral clearance (20). The previously utilized formalin-inactivated KFD vaccine was discontinued due to painful administration, waning immunity over time and the need for frequent booster doses (11, 12). Since no comparator vaccines are currently available on the market no comparative preclinical immune response studies were performed.

The protective efficacy of candidate vaccine was assessed in BALB/c mice, a well-established model for KFDV infection (23). Wildtype KFDV intraperitoneal infection in naive mice exhibited dose-dependent clinical manifestations, with mortality typically occurring around 9–11 dpi. Compared to earlier reports (23), the challenge strain demonstrated moderate virulence, as indicated by a higher lethal dose requirement and an extended median time to death. The KFD vaccinated mice conferred complete survival protection against high challenge dose of KFDV. Notably, this level of protection was sustained even five months post-vaccination, indicating the establishment of long-term immunological memory. While a single-dose regimen provided partial protection, the data clearly support the necessity of a two-dose schedule for achieving full protective efficacy. Although a single dose may offer limited initial efficacy, it holds potential for emergency use scenarios. KFD has two clinical phases including the primary phase wherein patients suffer from hematological abnormalities like leukopenia, thrombocytopenia, and elevated liver enzymes. In the secondary phase, the patients develop neurological complications (26). Similarly, the unvaccinated mice showed increasing viral load in the brain correlating with mortality around day 14 whereas, the vaccinated mice had no detectable virus in brain or spleen (**Figures 5A, B**). Confirms the vaccine’s ability to prevent neuroinvasive disease, a critical feature for KFDV, which can cause encephalitis-like illness (9). Vaccinated animals showed only a modest rise in nAbs post-challenge, (**Figure 5C**). Suggests that pre-existing nAbs (from vaccination) were sufficient for protection, whereas control animals only mounted a delayed response post-infection. Moderate anamnestic response in vaccinated mice implies pre-existing immunity prevented virus replication (27).

Neutralizing antibodies plays a crucial role in KFDV protection. Although the vaccine-induced nAb titers in mice were lower than those observed in live vector vaccine (23), the challenge experiments demonstrated clear and consistent protection against high-dose viral exposure. This finding is consistent with previous observations in Japanese Encephalitis (JE) vaccine studies, where protective efficacy was achieved even at relatively low antibody titers (28). Passive transfer of immune sera conferred the protection against lethal challenge in mice further supported the role of antibody-mediated immunity in KFDV protection as observed with live vector vaccine passive immunity study (23). Neutralizing antibodies were strongly associated with survival and are a key correlate of protection of flaviviral infections. Several animal studies demonstrated that nAb, induced by vaccination or passively transferred in serum, serve as a correlate of protection against flavivirus challenge (29–32). Although *ex vivo* neutralizing antibody titers in the current study indicated that nearly all mice with a titer of 10 or higher were fully protected (**Figure 8**), further comprehensive evaluation is necessary to establish the correlate of protection for the KFD vaccine.

Genetic variations within KFDV may influence vaccine effectiveness (2, 7, 13). The candidate vaccine demonstrated comparable neutralizing activity against lineages 2.1 and 2.2.1, suggesting that it is likely effective against currently circulating strains (**Figure 7A**). Shared antigenic features among flaviviruses can elicit cross-reactive immune responses, potentially affecting infection outcomes and vaccine efficacy (33, 34). Notably, the KFD vaccine immune sera (pooled) did not show cross-reactivity with major flaviviruses (**Figure 7B**). However, comprehensive screening for cross-reactivity remains essential, highlighting the need to evaluate responses against a broader range of flaviviruses. The role of antibody-dependent enhancement (ADE) in KFDV infection remains largely unexplored. In our study, the predominance of neutralizing antibody responses, together with the observed protection following KFDV challenge, suggests a low possibility of ADE, although further investigation is warranted. As a further step towards the clinical development, safety and immunogenicity of the KFD vaccine candidate evaluated in rodent and non-rodent animal models. Findings from *in vivo* observations, clinical pathology, necropsy data and histopathological evaluations have shown that the vaccine candidate demonstrated excellent safety profile in all the preclinical species tested.

In summary, intramuscular administration of KFD vaccine demonstrated an excellent safety profile. The vaccine also induced strong humoral and T cell immune responses and provided effective protection against challenge with the wild-type KFD virus. The KFD vaccine candidate has now received approval from the Drugs Controller General of India (DCGI) for advancement to a Phase 1 human clinical trial.

## Materials and methods

### Genomic analysis of KFDV from the year 1957 to 2022

To understand the diversity of KFDV strains and the antigenic variation among strains, genomic analysis was performed. For the study, 73 KFDV whole genome sequences were used from the 1957 to 1972-time period, as well as the 2006 to 2022 period submitted to GenBank by the Indian Council for Medical Research-National Institute of Virology (ICMR-NIV), Pune. The sequences were mapped against the reference genome of KFD (NC_039218) using reference-based mapping. Additionally, the consensus sequences of all genomes were extracted and aligned using the MAFFT standalone software. The phylogenetic analysis was carried out using the Maximum-Likelihood method in IQTREE2 using the substitution model with 1000 bootstrap replicates. The phylogenetic tree was visualized using Interactive Tree of Life. The variation in the E gene was also assessed. Low passage human isolates belonging to different lineages were chosen. The edge analysis of the isolates shortlisted were performed to rule out the presence of other human viruses like HIV, HBV, HCV and Mycoplasma.

### KFDV isolates adaptation in cells

Vero-CCL-81 cells were used for virus adaptation. The virus isolates selected based on genomic analysis were passaged five times in Vero-CCL-81 cells. For each passage, the virus titer was estimated using the plaque assay method. The genetic stability of the isolates across the passages were also assessed. RNA extraction was carried out using the standard protocol. Concentrations of extracted RNA were quantified using a Qubit 2.0 Fluorometer (Invitrogen, Carlsbad, CA, USA), and host ribosomal RNA was depleted using the NEBNext rRNA Depletion Kit (New England Biolabs, Ipswich, MA, USA). Quantified RNA was further used for the preparation of RNA libraries using the TruSeq Stranded mRNA Library Preparation Kit (Illumina, San Diego, CA, USA). The libraries were quantified using a KAPA Library Quantification Kit (Kapa Biosystems; Roche Diagnostics Corporation, Indianapolis, IN, USA) per the manufacturer’s protocol and loaded on an Illumina Miniseq next-generation sequencing platform. The libraries were normalized to 1nM, pooled together, and denatured using 0.1N NaOH. Further, the denatured libraries were neutralized using 0.1 M Tris (pH 7.0) and the libraries were further diluted to 1.8 pM using a hybridization buffer and loaded on an Illumina MiniSeq mid-output cartridge for paired-end sequencing. The FASTQ files generated after the completion of the run were analyzed using CLC Genomics Workbench software version 10.1 (CLC, Qiagen). The sequence of the cell-adapted virus (of 5 passages) was analyzed to confirm the genetic stability. The isolate which produced highest titer in cells were chosen for further studies.

### Viruses and cells

The KFD vaccine candidates used in this study were prepared by growing the KFDV strain no: NIV 164187 (GenBank Accession Number. MG720091; passaged in Vero CCL-81 (Passage 4) with titer of 10^7.5^/mL). The KFD seed virus was procured from the ICMR-National Institute of Virology (NIV), Pune, India. Vero WHO cells was used for vaccine development and manufacturing. BHK-21 cells were used for virus titration testing.

### Growth kinetics of KFDV

Vero cells were seeded in T-175 cm² flasks at a density of 0.15 × 10^5^ cells/cm² in EMEM supplemented with 10% FBS. The cells were incubated in a CO_2_ incubator at 36 ± 1 °C until reaching 80–90% confluency. Subsequently, the spent media was removed from the flasks, and the cell monolayer was washed with EMEM. The Vero cell monolayers in T-175 cm² flasks were infected with the research working virus of the KFD vaccine strain at multiplicities of infection (MOI) of 0.1, 0.01, or 0.001. The infected flasks were incubated in a CO_2_ incubator at 36 ± 1 °C. After 96 hours post-infection, the flasks from each MOI group were removed from the incubator and stored at -80 °C. The samples were later thawed and analyzed for virus titration.

### Manufacture of KFD vaccine candidate

Vero-WHO cells at a confluency of >85–90% were used for virus infection. The virus inoculum was prepared with 300 mL of EMEM per cell stack (ten-layer cell stack, CS-10), containing the required volume of virus as per the selected MOI. The infected cell stacks were then incubated at 36 ± 1 °C for approximately 96 hours until >90% cytopathic effect (CPE) was observed, characterized by cell rounding, detachment, and refractility. Harvests were collected into suitable sterile containers, the clarified KFDV harvest was subjected for inactivation through multiple downstream steps, endonuclease treatment, virus inactivation, clarification, molecular weight cut-off (MWCO) determination and chromatographic purification followed by sterile filtration.

### Dose escalation and efficacy studies

The inactivated vaccine of KFDV (Kyasanur forest disease virus) was formulated at different concentrations i.e. 6, 12, 18 and 24 µg/dose (0.5 mL) using two different adjuvants i.e. Aluminum phosphate and Aluminum hydroxide.

The 6-8 weeks old Balb/c mice were divided into 4 groups i.e. G1 to G4 and each group consists of 10 mice (5 male & 5 female). Further the G1, G3 and G4 groups were subdivided into four sub-groups such as G1A, G1B, G1C, G1D; G3A, G3B, G3C, G3D. The G1A, G1B, G1C and G1D group of mice were treated with Aluminum hydroxide formulation (Set-I) containing corresponding KFDV antigen concentrations at 6, 12, 18 and 24 µg/dose respectively, Similarly G3A, G3B, G3C and G3D group of animals were treated with Aluminum phosphate formulation (Set-II) containing 6, 12, 18 and 24 µg/dose of KFDV antigen. The G2 and G4 groups were included as respective Aluminum hydroxide and Aluminum phosphate adjuvant placebo control groups. All the groups of mice were immunized on Day 0 and 28 by intramuscular route at a dose of 0.5 mL. The animals were bled at day 0e and post-immunization on Day 21, 42, 60, 90, 120 & 150 collected sera subjected for binding and neutralizing antibody assessment by ELISA and PRNT respectively. For vaccine efficacy assessment, vaccinated and placebo animals (n=30) were subsequently, challenged intraperitoneally with 10-1000 LD_50_ (log_10_ 3.38 to 5.38 PFU) of KFDV. Infected animals were monitored for 42 days post-challenge. For viral load assessment studies, both vaccinated and placebo groups (n=20) were challenged intraperitoneally with 100 LD_50_ (log_10_ 3.38 to 5.38 PFU) of KFDV. Subset (n=4) mice from each group were euthanized for sample collection on days 5, 8, 11, 14, 17, 21 and 42. Surviving mice were monitored until 42 days post-challenge.

### Clinical scoring

BALB/c mice were exposed to 10, 100, or 1000 LD_50_ of KFDV corresponding to approximately 3.38, 4.38 and 5.38 pfu/0.2mL respectively. Animals were observed twice daily for the appearance of clinical symptoms or any abnormal changes. A standardized clinical scoring system (shown in the table below, **Supplementary Table 1**) was used to evaluate animal health throughout the observation period. Each score reflects defined physical signs and associated percentages of body weight loss, allowing assessment of disease progression and identification of humane endpoints. Clinical manifestations—including weight loss, hunched posture, ruffled fur, and reduced activity—appeared between 7- and 9-days post-infection (dpi), depending on the viral dose administered. Mortality was typically observed around 10–11 dpi.

### Passive immunization and passive transfer study

Groups of 20 BALB/c mice (6–8 weeks of age) were immunized on Day 0 and 28 by intramuscular route at a dose of 0.5 mL. On day 42 blood was collected from all mice. Sera from all vaccinated and placebo animals were pooled and the IgG and neutralizing antibody titer was determined by ELISA and PRNT respectively. Groups of 10 BALB/c mice were administered IP with 500µl of KFDV or placebo immune sera. Mice were challenged with 10 LD_50_ of KFDV 24 later. Of these, 6 mice were allocated to the survival monitoring cohort and were followed for clinical signs and mortality for 42 days post-challenge. The remaining subset of mice (n=4) were assigned to the tissue collection and were euthanized at the specified time point day-11 post-challenge for viral load testing.

### Enzyme linked immunosorbent assay

The KFDV whole virion antigen was diluted in carbonate-bicarbonate buffer (pH 9.6) to approximately 2.5 µg/mL. The resuspended antigen of 100 µL per well was added to a 96-well plate and the plates were incubated overnight at 2–8 °C for adsorption. The unbound antigen solution was discarded, and the plate was washed four times with PBS-T. Blocking buffer (200 µL per well) was added to each well, and the plate was incubated for 1 hour at 37 °C. Test sera were diluted (e.g., 1:100 starting dilution up to 1:12800, with serial 2-fold dilutions). A volume of 100 µL per well of diluted sera was added, and the plates were incubated for 1 hour at 37 °C. The plate was then washed four times with PBS-T. Horseradish Peroxidase (HRP)-conjugated anti-IgG was diluted as per the manufacturer’s instructions (typically 1:10,000). A volume of 100 µL per well was added, and the plate was incubated for 1 hour at 37 °C followed by PBS-T washes (four times). Tetramethylbenzidine (TMB) substrate (100 µL per well) was added to all wells, and the plate was incubated in the dark for 15 minutes at room temperature. Stopping solution (100 µL per well) was then added, and the optical density (OD) was measured at 450 nm using a plate reader.

### Plaque reduction neutralization assay

Animals were sacrificed and serum was collected for the PRNT assay. The serum samples were heat-inactivated at 56 °C for 30 min prior to test. Twofold series dilution was performed starting from 5-fold. 24-hrs old BHK21 cells seeded on 6 well plates were used for the PRNT test. The diluted serum was then combined with the 1000 PFU/ml KFDV live virus at a 1:1 ratio for neutralization and incubated at 37 °C for 1.5h. The virus-sera mixture is then inoculated onto BHK 21 cells and left for virus adsorption at 37 °C for 1.5h in 5% CO2 conditions. After 90 minutes, the cells are topped up with 2mL of overlay medium containing 1.5% methyl cellulose and the plates are further incubated for 4 days at 37 °C in 5% CO2 conditions. After 4 days, the plates were removed from the CO2 incubator, washed with PBS and stained with 0.5% crystal violet solution. After 20 min, the crystal violet solution is discarded, washed and kept for drying. The plaques are observed as hallows against black background cell monolayer and the PRNT_50_ titer was estimated using the Spearman-karber method. The PRNT titer is defined as the % reduction observed from the count of plaques in the virus control. Therefore, PRNT_50_ is the dilution of serum which can neutralize 50% of the no. of plaques observed in the virus control.

### Cross-neutralization assay

Cross-reactivity of KFD polyclonal sera was checked with other flaviviruses such as JEV, DEN-2, and Zika. Known positive KFD sera were initially diluted to 1:5, followed by 2-fold serial dilution and 1000 PFU/mL of viruses KFDV (NIV-164187), DEN-2 (wildtype isolate), JEV B-05 and Zika (MR766) viruses were added in equal volumes. Neutralization was performed for 1.5 h at 37 °C with 5% CO_2_. Following incubation, the neutralized virus-sera were transferred to 24 h old BHK21 cell-seeded plates and incubated for 1.5 h at 37 °C with 5% CO_2_ and overlaid with 1.5% MC and incubated for 4 days and the PRNT_50_ titer was estimated using the Spearman-karber method.

### Cell-mediated immune response

On day 69, following secondary immunization, spleens were aseptically collected from the immunized mice. Splenocytes were isolated and seeded at a density of 3 × 10^5^ cells per well in 96-well flat-bottom plates (Nunc) containing 200 µL of RPMI-1640 medium (Gibco) supplemented with 10% fetal bovine serum (FBS). Cells were stimulated with KFDV recombinant envelope (rE) protein at a concentration of 1 µg/mL and Concanavalin A (ConA) at 2.5 µg/mL. The cultures were incubated at 37 °C in a 5% CO_2_ atmosphere for 48 h. Following incubation, proliferative cellular immune responses were assessed using the specific cytokine assay kits. After the incubation period, supernatants from each well were collected, and ELISA was performed to measure the concentrations of IFN-γ, TNF-α, IL-6, IL-4, and IL-10 using cytokine ELISA kits according to the manufacturer’s instructions (R&D Systems). Cytokine levels were calculated using standard curves generated with known concentrations of the recombinant cytokines. The results were expressed in picograms per milliliter (pg/ml).

### KFDV bioburden determination

To assess the viral load following secondary immunization, brain and spleen samples were collected from experimental animals on days 5, 8, 11, and 14 post-challenge. Each collected brain and spleen tissue was mechanically homogenized (triturated) using a sterile homogenizer to obtain a uniform suspension. The homogenate was clarified by centrifugation at 3000 × g for 10 min at 4 °C, and the supernatant was collected for viral quantification.

The presence of infectious virus in the tissue samples was determined using a plaque assay on BHK-21 cell monolayers. Briefly, BHK-21 cells were seeded in 6-well plates 24 h prior to infection to achieve 90–100% confluency. Serial 10-fold dilutions of the tissue supernatants were prepared using MEM. A volume of 200 µL from each dilution was added to the wells in duplicate and allowed to adsorb for 1.5 h at 37 °C with gentle rocking every 15 min. After adsorption, the inoculum was removed, and the wells were overlaid with 1.5–2 mL of 1.5% carboxymethylcellulose (CMC). Plates were incubated at 37 °C in a 5% CO_2_ incubator for 4 days. The monolayers were stained with 1% crystal violet solution for 15–30 min, rinsed with distilled water, and air-dried. Plaques were counted with the naked eye, and viral titers were calculated as plaque-forming units per milliliter (PFU/mL) of the original tissue homogenate, adjusted for dilution factors.

### Quantification and statistical analysis

Statistical analysis GraphPad Prism version 10 (GraphPad Software, San Diego, CA) was used to plot all the graphs and statistical analysis of the data.

## Data Availability

The original contributions presented in the study are included in the article/**Supplementary Material**/online repositories. The genomic data can be found here: [https://www.ncbi.nlm.nih.gov/nuccore/MG720091.1]. Further inquiries can be directed to the corresponding author.
