# Supplementary material for: Development of whole virion inactivated Kyasanur Forest Disease vaccine candidate and its preclinical safety and efficacy evaluation
Source: Front Immunol. 2026 Apr 2;17:1786057. doi: 10.3389/fimmu.2026.1786057 (PMC13083075; doi:10.3389/fimmu.2026.1786057)
Supplement: Supplementary file 1 [file Table1.docx]

**Supplementary Material**

**Development of whole virion inactivated Kyasanur Forest Disease vaccine candidate and its preclinical safety and efficacy evaluation**

Raju Sunagar^a^*, Sreelekshmi Mohandas^b^, Narayana Penta^a^, Rekha Cheruvara^a^, Rama Lakshmi Bonda^a^, Praveen Algangula^a^, Virendra Kumar Meena^b^ Sathish Kumar, Sridevi Nimmagadda, Naveen Kumar^b^, Nivedita Gupta^C^ Pragya Yadav^d,b^, Anand Kumar Kanakasapapathy^a^.

^a^Indian Immunologicals Ltd, Rakshapuram, Gachibowli Post, Hyderabad 500032, India.

^b^Indian Council of Medical Research-National Institute of Virology, Pune 411001, India.

^c^Indian Council of Medical Research, Head Quarter, New Delhi 110029, India

^d^Indian Council of Medical Research-National Institute of One Health, Nagpur 411021, India.

*Corresponding author-Raju Sunagar, [s.raju@indimmune.com](mailto:s.raju@indimmune.com)

# **Pre-Clinical Toxicity Studies**

During the development of new vaccine, the purpose of pre-clinical studies is to provide safety information to support the clinical development and licensure of the product. The Preclinical study designs had taken into account the intended clinical use, route of administration, dose levels and immunization schedule in humans. Pathology phase of repeated dose toxicity studies were performed at Vivo Bio Tech limited, Hyderabad, which is an OECD GLP certified and AAALAC accredited facility as per Schedule Y, ICH and WHO guidelines following GLP standards.

## **Test item.** Inactivated KFD vaccine candidate was used for the pre-clinical studies which represented the vaccine formulation that is intended for clinical use. The test item was administered at three doses viz., 9 mg/animal, 18 mg/animal & 36 mg/animal as low dose (0.5X human dose (HD)], mid dose (1X HD) and high dose (2X HD), respectively. A placebo group administered with vaccine excipients served as control group. Recovery groups for placebo, (1X HD) and (2X HD) doses were also maintained in the studies.

## **Test system.** The test system *viz*., Wistar rats, Swiss webster mice and New Zealand White rabbits were sourced from Committee for the Purpose of Control and Supervision of Experiments on Animals (CPCSEA) approved vendor and these test systems were selected as per the recommendations of WHO guidelines (2013) and Schedule Y (2019). Young adult animals were used and the average body weight ranged from 157 - 250 g for Wistar rats, 11 - 17 g for Swiss webster mice and ~ 2 kg in both males and females for New Zealand White (NZW) rabbits. The studies were conducted in equal number of male and female animals.

## **Animal Husbandry Practices.** The animal rooms were air-conditioned with adequate air changes (12 - 15) per hour and provided with a light cycle of 12 hours light and 12 hours dark. The room temperature was maintained in the range of 22 ± 3°C (rat and mice), 20 ± 3°C (rabbits) and relative humidity between 30 and 70% and it was continuously monitored. Animals were housed in cages with dimensions adhering to CPCSEA guidance. Maintenance Diet and UV Purified water were provided *ad libitum*.

## **Ethical Approvals.** The studies were conducted post approval from Institutional Animal Ethics Committee (IAEC) and Institutional Biosafety Committee (IBSC) following all ethical practices as laid down in the guidelines for animal care and biosafety.

## **Treatment regimen.** The dose volume of 500 µL/animal (split dose; 50 µL/nostril) was administered by intranasal route in the four *in vivo* models on day 0, 21 and 28 (N+1) with full Human Single Dose (HSD)**.**

## **Dose Administration.** Site of injection was wiped with 70 % Isopropyl alcohol before dose administration. The test item of low dose (0.5X HD), mid dose (1X HD) and high dose (2X HD) was administered to the G2, G3 & G6 and G4 & G7 respectively by intramuscular route on Day 0, 14 and 28 (N+1). Similarly, the placebo was administered to the G1 & G5 (placebo) group of animals. The main group animals from placebo, mid- and high-dose groups were sacrificed 2 days, low-dose animals were sacrificed 7 days, and recovery groups were sacrificed 14 days post last dose. The total 0.5 mL dose volume of test item/placebo was administered intramuscular using suitable syringe attached to 26-gauge needle. The injections 1 and 3 were injected to left flank and injection 2 were injected to right flank of corresponding group of rabbits at defined time intervals on Day 0, 14 and 28. The site of injection on each animal was marked with indelible ink for identification at necropsy and the injection sites were marked as often as needed to assure the identification.

## **Clinical Pathology.** Blood samples were collected at predose on Day -2, post immunization on Day 3 (for main and recovery group of animals) and, prior to terminal sacrifice on day 32 from main group of animals and on day 43 from recovery group of animals. Animals were fasted overnight for a period of approximately 12 - 18 hours prior to blood collection (water was provided *ad libitum* during fasting). Blood samples were withdrawn through central ear artery/ marginal ear vein for hematology and clinical chemistry analyses.

## **Statistical Analysis.** All continuous data like body weight, body weight gain, Food consumption, rectal temperature, hematology, clinical chemistry, absolute organ weights and relative organ weights were subjected for various statistical analyses using software “SYSTAT Version No. 13”. Data was evaluated by ANOVA. The parameters were analyzed at 5% (p<0.05) level of significance.

**Results**

**Figure. S1** Envelope ectodomain specific binding antibody levels. ). Six to eight-week-old BALB/c male and female mice were immunized with various doses of KFD vaccine or placebo (n = 10) via intramuscular route. Binding and neutralizing antibody responses at 2 weeks post-vaccination (day 42) in sera of immunized mice at various doses of KFD vaccine or placebo. Binding antibody levels against Inactivated whole virion KFDV or recombinant envelope protein ectodomain [Nordic Biosite, BTF38GD8] were measured by ELISA. Statistical significance for binding antibody titers was performed with parametrict unpaired t test.


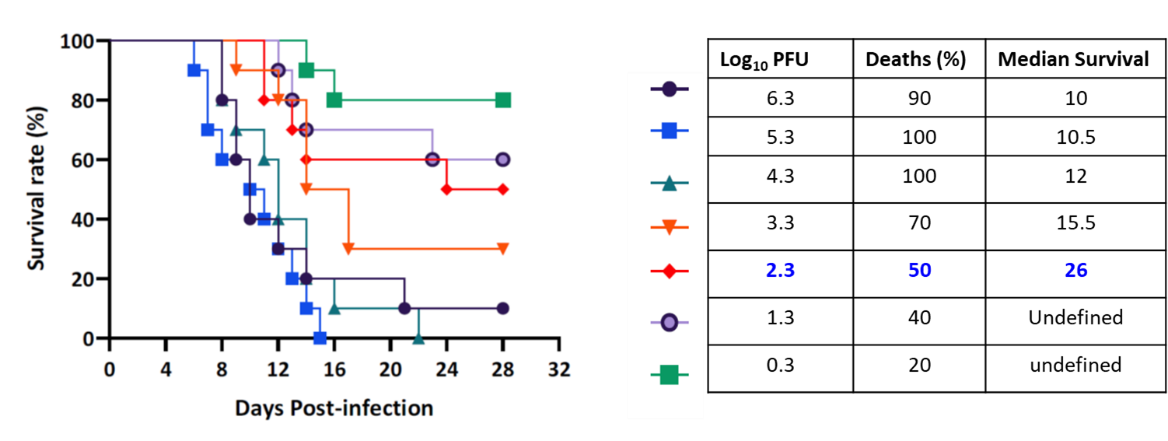


**Figure. S2** **Determination of median lethal dose (LD_50_) in BALB/c mice.** The KFD challenge virus (Stock virus log titer: 10^6.3^ pfu/0.2 mL) was serially diluted from 10^-1^ to 10^-6^ - fold using sterile PBS as a diluent. The undiluted neat and dilutions of challenge virus from 10^-1^ to 10^-6^ were inoculated intraperitoneal (0.2 mL/mice) to the individual groups of mice (n=10). Post inoculation all the groups of mice were observed for KFD disease symptoms body weight, clinical signs and survival rate of mice were recorded. The survival rate of mice was observed till Day 28 and the LD_50_ of challenge virus dose was calculated using Redd & Muench method.

**Figure S3. Protective efficacy of various doses of the KFD vaccine against lethal challenge with wild-type KFD virus**. Six to eight-week-old BALB/c male and female mice (n=10) were immunized with various doses of KFD vaccine or placebo (n = 10) via intramuscular route on days 0 & 28 and challenged on day 42 post-vaccination. Challenged animals were monitored for 42 days following lethal challenge with wild type KFDV with 100 LD_50_.

**Figure. S4** Single dose-Day 28 challenge. six-eight-week-old BALB/c male and female mice were immunized with single dose vaccination subjected for wild type KFDV challenge on day 28 post-vaccination, mice were monitored for weight-loss and survival for 42 days.

**Figure. S5** Single dose-Day14 challenge. six-eight-week-old BALB/c male and female mice were immunized with single dose vaccination subjected for wild type KFDV challenge on day 14 post-vaccination, mice were monitored for weight-loss (A) and survival (B) for 42 days. Statistical significance for survival curves was analysed using the Lograng (Mantel-Cox) test.

**Figure. S6** Immunnogenicity in rat, rabbit (A & B) and hamster (C) blood samples were withdrawn through central ear artery from all the main and recovery group of rat and rabbits on day -2 (pre-dose) and post-immunization from all the group of animals on day 25 and the terminal bleed day 42. Binding antibodies were measured in serum samples against Inactivated whole virion KFDV antigen. Six to eight-week-old Syrian hamster (male and female) were immunized with KFD vaccine or placebo (n = 10) via intramuscular route. Binding and neutralizing antibody responses at 2 weeks post-prime & boost vaccination (day 21 & 42). Statistical significance for binding and neutralizing antibody titers was performed with parametrict paired t test and unpaired t test with Welch’s correction respectively.

**Table S1**. Clinical scoring system for animal health assessment

| **Score** | **Indications** | **Weight loss %** |
| --- | --- | --- |
| 1 | Ruffled fur | < 10 % |
| 2 | Ruffled fur/hunched posture | < 10 % |
| 3 | Ruffled fur/hunched posture/lethargy | < 15 % |
| 4 | Ruffled fur/hunched posture/lethargy/paresis | < 15 % |
| 5 | All of the above plus ≥15% weight loss (humane endpoint) | ≥ 15 % |
